# A review on generative AI models for synthetic medical text, time series, and longitudinal data

**DOI:** 10.1038/s41746-024-01409-w

**Published:** 2025-05-15

**Authors:** Mohammad Loni, Fatemeh Poursalim, Mehdi Asadi, Arash Gharehbaghi

**Affiliations:** 1 School of Innovation, Design and Engineering, Mälardalen University, Västerås, Sweden; 2 Servicehälsan Familjeläkare i Västerås AB, Västerås, Sweden; 3 ICT Department, Turku University of Applied Sciences, Turku, Finland; 4 Department of Biomedical Engineering, Linköping University, Linköping, Sweden

**Keywords:** Health care, Medical research

## Abstract

This paper presents the results of a novel scoping review on the practical models for generating three different types of synthetic health records (SHRs): medical text, time series, and longitudinal data. The innovative aspects of the review, which incorporate study objectives, data modality, and research methodology of the reviewed studies, uncover the importance and the scope of the topic for the digital medicine context. In total, 52 publications met the eligibility criteria for generating medical time series (22), longitudinal data (17), and medical text (13). Privacy preservation was found to be the main research objective of the studied papers, along with class imbalance, data scarcity, and data imputation as the other objectives. The adversarial network-based, probabilistic, and large language models exhibited superiority for generating synthetic longitudinal data, time series, and medical texts, respectively. Finding a reliable performance measure to quantify SHR re-identification risk is the major research gap of the topic.

## Introduction

Application of digital technologies, e.g., artificial intelligence, to improve medical management, patient outcomes, and healthcare delivery, is known as digital medicine^1,2^. This context has significantly evolved towards intelligent decision-making after the development of deep learning (DL) models. A common feature of most DL models is the need for a large dataset for training and validation of the models. Preparing a large dataset that incorporates sufficient samples of various classes for training a DL model is sometimes problematic. This is particularly evident when it comes to medical data in which the privacy of patient data demands serious attention.

Social and medical communities rigorously assign escalating regulations at the different societal levels to avoid misconduct of patient data. In this light, the European Union has recently released the first regulation^3^ on artificial intelligence towards lowering the risk of data abuse and further governance on the developers. Such pertinent restrictions as well as difficulties in collecting medical data, act as the impeding factors in preparing a sufficiently large and fair dataset for training and validation of the DL models. Medical data is nowadays stored in a digital fashion, named Electronic Health Record (EHR), which is composed of a set of the health-related data collected from the individuals of a population during their visits to any care unit defined by the healthcare system of the population^4^. An EHR typically contains demographic data, clinical findings, lab values, procedures, medications, symptoms, diagnoses, medical images, physiological signals, and descriptive texts obtained from the individuals during the visits. During a visit to a care unit, and depending on the visit, an individual may undergo a sequence of investigations and/or examinations, called events, which are stored in the EHR of the individual, using the globally known codes, e.g., international classification code (ICD)^5^ for the diagnosis. A sequence of the tabular health records of an individual, resulting from several visits, constitutes longitudinal health data^6,7^.

Accessibility of the EHR can be of vital importance when it comes to the patient management, and hence, highest level of security considerations are administered to preserve privacy of EHRs. Healthcare systems set intensive restrictions and limitations for accessing EHR contents. As a result, preparing a rich clinical dataset is considered as an important research question for any study in the digital medicine context. A practical solution to this research question is to create a synthetic copy of the clinical dataset from the real one, and use it for research purposes. The created dataset, which is called synthetic health record (SHR), can be utilized and shared with the researchers, instead of the real ones, for the training and validation of DL models. A SHR is created to resemble the general characteristics of EHR data while ensuring the subjects of the EHR remain unidentifiable^8–10^. Such datasets can be employed by any machine learning method for training and optimization purposes.

Health records can incorporate tabular data of the medical records, longitudinal tables of different visits, time series of physiological signals, textual clinical data, and medical images. Recent progress in developing DL models for generating various modalities of synthetic data enabled researchers to address the diverse research gaps that existed in the preparation of high-quality health data. Topics such as privacy leakage, limited data availability, uneven class distribution, and scattered data, are regarded as some of the research topics of this domain.

Despite substantial progress in developing generative models for synthetic medical data, there is still a big room for studying the reliability of the generated data. As we will see in the discussion section, the reliability of the models is explored from two different perspectives: the quality of generated data in terms of conformity with real data, and the capability of learning models to preserve the privacy of the real data in terms of re-identification. Discrepancy in the study objectives for various data modalities, and inconsistency in the evaluation metrics, make it a complicated task to select an appropriate model for creating SHR along with a realistic evaluation of the model.

This paper presents the results of a scoping review study on the existing DL models for generating SHRs. The review examines the DL models deployed, the modalities utilized, the datasets invoked, and the metrics employed by the researchers to explore the scope and potential of using SHR for different medical objectives. The study taxonomy is performed in both technical and applicative manners to represent the relevance of the models in conjunction with their capabilities in producing time series, text, and longitudinal SHR. The main study objectives are the identification of the practical capabilities and the knowledge gaps of generative models for creating SHR, which are detailed as follows:

- Finding state-of-the-art of the generative models for creating synthetic medical texts, time series, and longitudinal data, along with the methodological limitations.
- Summarizing the existing performance measures in conjunction with the related metrics for evaluating the quality of SHR.
- Listing the most used datasets employed by the researchers for generating SHR.
- Finding the key research gaps of the field.

The unique features of this study are mainly the comprehensiveness of the review and the novel research taxonomy. As we will see in the discussion section, this paper introduces innovative features compared to the other review papers. These features initiate the following contributions to the field:

- Introducing taxonomic novelty by defining various data modalities and applications. There are review papers on SHR for tabular and image data, however, less attention was paid to important topics such as medical texts and longitudinal data.
- Evaluation of different machine learning (ML) methods for generating various forms of SHR.
- Representing the performance measures and the evaluation metrics used for validating the quality of SHR in association with the related methodological capabilities for evaluating different data modalities.
- Introducing available datasets for generating SHR in conjunction with the related applicability per the study taxonomy.

It is worth noting that the number of publications in the SHR domain has drastically increased in the last two years. The novel aspects of this study project the scope and the possibilities of state-of-the-art methods in this new domain of digital medicine.

## Results

### Overview of the findings

**Fig. 1:**
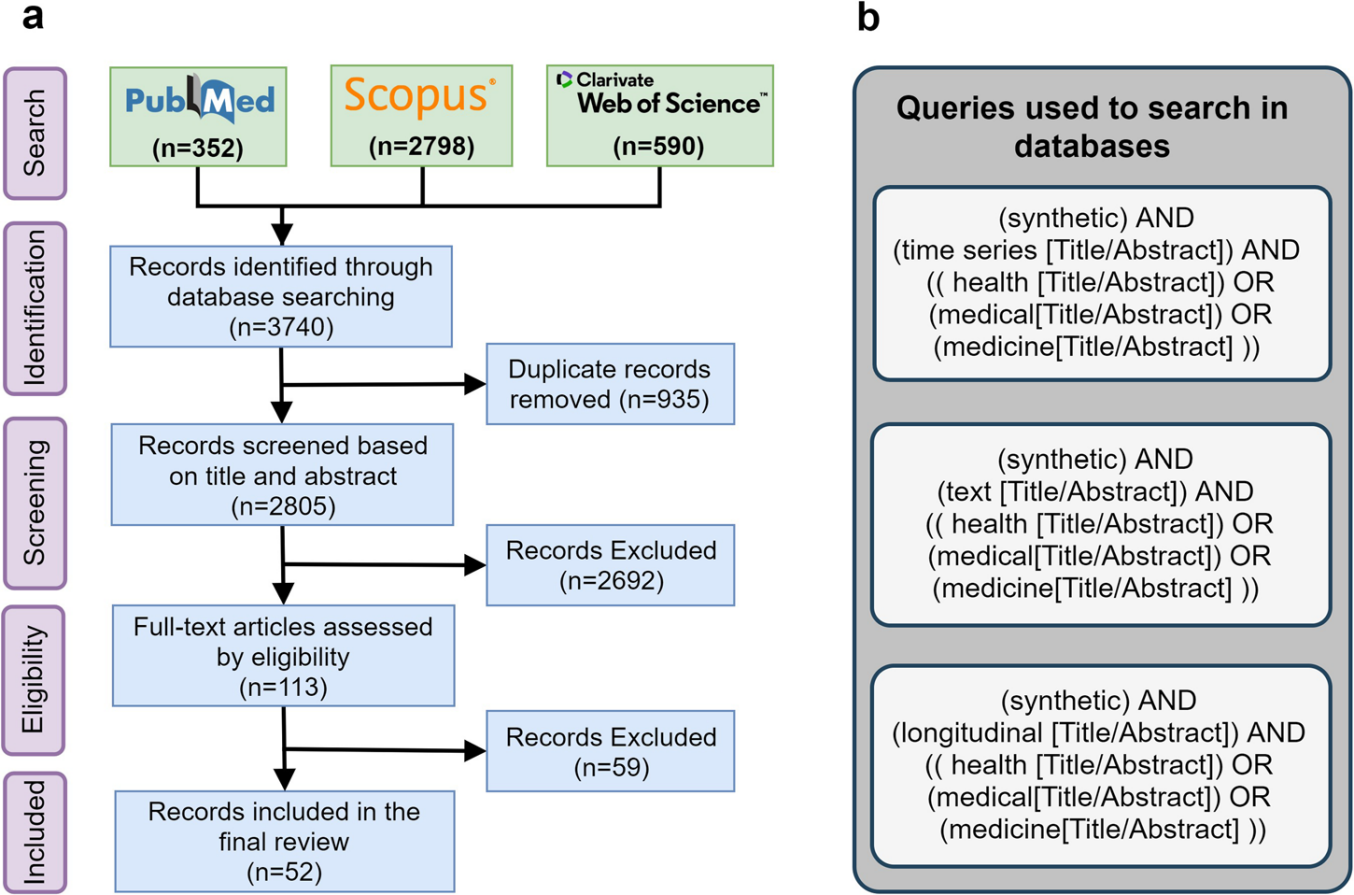
Overview of the study selection process and research queries. **a** PRISMA-ScR representation of the research methodology. **b** Research queries used in the study.

Figure 1a illustrates an overview of the identified publications. In total, 3740 citations resulted from the bibliographic search in PubMed (*n* = 352), Scopus (*n* = 2798), and Web of Science (*n* = 590) in the Identification phase, from which 935 of the citations were excluded due to duplication. In the screening phase, after carefully reading Tile and the Abstract of the publications, 2692 publications were filtered out because of the topical irrelevance. In the Eligibility phase, 52 publications fulfilled the inclusion and exclusion criteria and ultimately participated in the study (PubMed = 27, Scopus = 19, and Web of Science = 6). Note that half of the eligible publications (*n* = 26) were interestingly published after 2022. Additionally, we included the related review or survey papers, published between 2022 and 2023 (Table 4). All of the included review papers were articles in peer-reviewed journals.

**Fig. 2:**
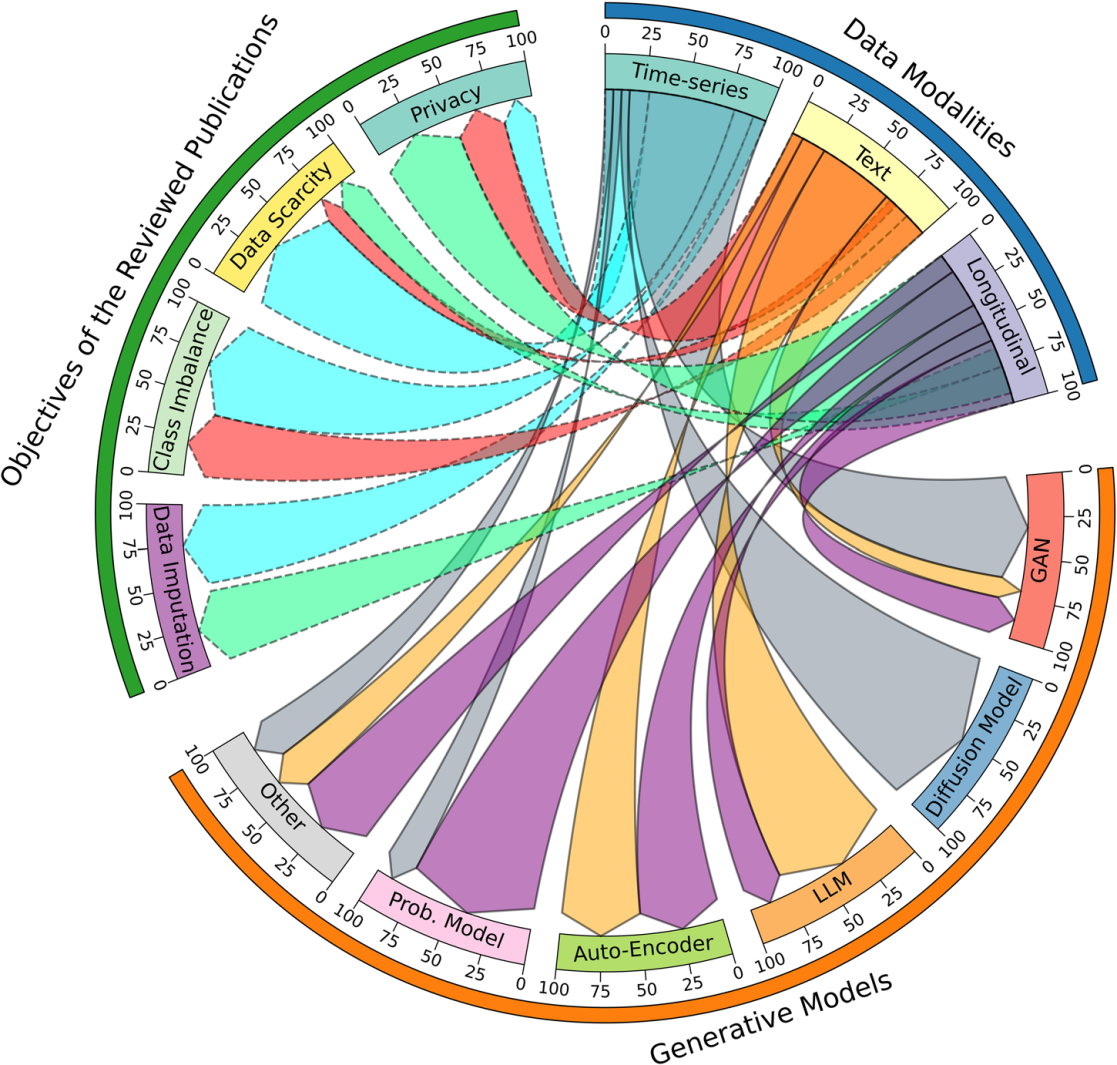
The mutual link between the data modalities, generative models, and research objectives found by the research objectives. This Figure shows an overview of the findings in the paper. In summary, generative adversarial networks (GANs) were dominantly employed for generating medical time series. Large language models (LLMs) have been widely used for generating synthetic texts. The variational auto-encoder (VAE) method was employed in a minority of the studies, equally for generating longitudinal and text data, but it was not used for time series. Probabilistic models were dominantly utilized for longitudinal data.

Figure 2 represents an overview of the findings. It is observed that methods based on generative adversarial networks (GANs) were dominantly employed for generating medical time series, and much less, for generating the longitudinal and the text modalities, respectively. The diffusion model was merely used for this data modality. Although large language models (LLMs) have been well-received mainly for generating synthetic texts, their application in generating longitudinal data showed promising results. The variational auto-encoder (VAE) method was used in a minority of the studies, equally for generating longitudinal and text data, but not so for the time series. The probabilistic models, e.g., Bayesian network, were mostly used for the longitudinal data, and trivially for the time series.

### Medical time series data

Generation of synthetic physiological time series was observed in 22 reports (42%) of the published peer-reviewed papers, from which synthetic Electrocardiogram is the most common case study (10 studies). Electroencephalogram was the main topic of the second most common objective where the diffusion model resulted in the optimal utility^11^. Data scarcity and privacy were found to be the main objectives of 12 and 6 studies, respectively. Class imbalance and imputation constitute the objective of the rest of the 4 studies. A great majority of the studies relied on the various fashions of the GAN-based methods where statistical models, such as the diffusion model and hidden Markov model were seen in a minority of 10% of the studies. Table 1 lists the findings of the survey on the generative models for synthetic time series.

**Table 1:** The reviewed publications on generating synthetic medical records of time series

Paper & Year	Study Objective	Case Study	Method	Key Takeaways
^11^, 2023	Privacy	Normal & abnormal ECGs	Diffusion model	(+) Outperforming GAN-based competitors by capturing long-term dependencies
^34^, 2023	Data scarcity	Non rapid eye movement sleep Electroencephalogram (EEG)	cGAN	(-) Lack of proper evaluating the fidelity of generated data
^12^, 2023	Data scarcity	Post-stroke assessment	Time series siamese GAN	(+) Generating heterogeneous data by avoiding mode collapse
^46^, 2023	Data scarcity	Electrocardiogram (ECG) arrhythmia, normal & abnormal ECGs	Time series GAN	(-) Requiring additional experiments to determine the optimal hyperparameters of long short-term Memory (LSTM)
^47^, 2023	Data scarcity	Human physical activities	Temporally correlated multi-modal GAN	(+) Preserving temporal correlation between variables learned from multi-modal irregular data
^48^, 2023	Data imputation	Bivariate time series of arterial blood pressure and ECG	Recurrent cGAN	(+) Handling missing time series with different sizes and positions
^17^, 2023	Class imbalance	ECG record with paroxysmal atrial fibrillation	GAN	(+) Using physician knowledge to verify generated synthetic samples
^49^, 2022	Data scarcity	Activity recognition, simulated sinusoidal waves, normal & abnormal ECGs	Transformer-based time series GAN (TTS-GAN)	(+) Effective handling very long time series
^50^, 2022	Data scarcity	Parkinson disease, human physical activities	Multi-axial cGAN (SensoryGANs)	(+) Synthesizing long multi-axial wearable sensor data
^51^, 2022	Data scarcity	Venous leg ulcer, arterial ulcer, diabetic foot ulcer	Time series conditional Wasserstein GAN	(+) Eliminating the need for access to a big EHR dataset
^52^, 2022	Privacy	Intensive care unit (ICU) records (oxygen saturation, heart rate, respiratory rate, etc.) in the MIMIC III database	Time series GAN (HealthGen)	(+) GAN provides better results compared to RNN and Kalman VAE
^53^, 2021	Privacy	Normal sinus rhythm (NSR), ECG arrhythmia	Neural ordinary differential equation GAN	(-) Lack of quantitative evaluations
^13^, 2021	Privacy	NSR, ECG arrhythmia	Multivariate GAN	(+) Avoiding GAN mode collapse
^54^, 2021	Data imputation	EEG with sleep stage labels	GAN	(+) Preserving contextual latent in formations
^55^, 2021	Class imbalance, data scarcity	NSR, ECG arrhythmia, EEG data on genetic predisposition to alcoholism	Guided evolutionary synthesizer	(+) Using a non-differentiable objective function
^15^, 2020	Data scarcity	ICU records (oxygen saturation, heart rate, respiratory rate, etc.)	LSTM-based controllable GAN with spectral normalization	(+) Avoiding mode collapse with spectral normalization (-)
^56^, 2020	Data scarcity	Human physical activities (walk, jog, etc.)	GAN	(-) Evaluations are limited to visualization checks
^57^, 2020	Class imbalance	Photoplethysmogram (PPG) abnormal	cGAN	(+) Evaluating several cGAN model
^14^, 2020	Privacy	ECG arrhythmia	Multivariate GAN	(+) Using a mini-batch discrimination layer for avoiding mode collapse
^58^, 2020	Privacy	EEG, ICU records (oxygen saturation, heart rate, respiratory rate, etc.)	Conditional & Temporal GAN (PART-GAN)	(+) Integrating differential privacy paradigms with generating noisy and irregularly-sampled time series
^16^, 2019	Data scarcity	Obstructive sleep apnea	Concatenating multiple GAN models	(+) Avoiding mode collapse by concatenating results of multiple GANs
^59^, 2019	Data scarcity	Smart home-based activity data	Combination of hidden Markov model (HMM) and regression algorithm (SynSys)	(+) Benefiting from HMM’s sequence-generative nature

### Medical longitudinal data

Table 2 lists the survey’s findings on the generative models for synthetic longitudinal data. As can be seen, privacy is the main objective of the 16 out of the 17 studies with various case studies comprising kidney diseases, patients with hearing loss, Parkinson’s and Alzheimer’s diseases, chronic heart failure disease, diabetes, hypertension, and hospital admissions. The GAN-based methods were observed in the great majority of the studies as the optimal model outperforming the baseline studies.

**Table 2:** The reviewed publications on generating synthetic medical records of longitudinal data

Paper & Year	Study Objective	Case Study	Method	Key Takeaways
^60^, 2023	Privacy, data scarcity	Hospital visits from MIMIC III database	Hierarchical auto-regressive language model	(+) Fidelity of SHR is improved by utilizing a probabilistic and an autoregressive model for estimating longitudinal data at the visit and code level
^61^, 2023	Data scarcity, privacy	multi-dimensional cancer and type-2 diabetes data	GAN-boosted semi-supervised learning	(+) Utilizes the underlying graphical structure of EHRs
^6^, 2023	Privacy, data scarcity	EHR time series for ICU patients	Mixed-type longitudinal GAN	(+) Generating mixed-type time series by effectively capturing the temporal characteristics of the original data
^9^, 2023	Privacy	Critical care patients data admitted to ICU (e.g., #visits, diagnosis) from MIMIC IV dataset	Variational graph auto-encoder	(+) Generating synthetic patient trajectories from EHRs with graph learning
^7^, 2023	Privacy	Longitudinal health records (e.g., age, vital statistics)	RNN	(-) Generating lengthy sequences has limitations
^35^, 2022	Data scarcity	Type-2 diabetes data	Generative Markov-Bayesian-based model	(-) limited to a single chronic disease and using only ICD-10 data code
^62^, 2022	Privacy	Health records of patients with hypertension	GAN	(-) The criteria for data inclusion and exclusion could potentially result in selection bias
^63^, 2022	Privacy, data imputation	Parkinson’s disease and Alzheimer’s disease	Multi-modal Neural Ordinary Differential Equations	(+) Handling multi-modal data along with learning continuous-time real data trajectories (-) Limited to the static categorical variables
^64^, 2022	Privacy	Hospital visits from MIMIC III database	GPT-2	(+) Formulating the generation of the heterogeneous EHRs as a text-to-text translation task using LLMs
^65^, 2022	Privacy, data imputation	Hospital visits from MIMIC III database	DataSifter-II (ruled-based method)	(+) Improved privacy of the time-varying correlated data by using a generalized linear mixed model and random effects-expectation maximization tree
^8^, 2021	Privacy	Hospital visits from MIMIC III database	Bayesian network	(-) Struggling to preserve multivariate relationships in the datasets
^66^, 2021	Privacy	Acute kidney injury	GAN	(-) Insufficient evaluation of the fidelity and the utility
^67^, 2021	Privacy	The EHR from type-2 diabetes, heart failure, and hypertension	GAN	(+) Mitigation of the GAN issues by using a two-step learning method: dependency learning and conditional simulation
^36^, 2020	Privacy	Hospital visits from MIMIC III database	Adversarial auto-encoder	(+) Adversarially learning both the continuous latent distribution and the discrete data distribution
^68^, 2020	Privacy	Chronic heart failure, organ transplantation	cGAN	(+) Improved privacy; the identifiability of the SHR is quantified and employed for the optimization of a cGAN
^69^, 2019	Privacy	Hearing loss patients	Bayesian network	(-) Insufficient evaluation of the fidelity and the utility
^70^, 2019	Privacy	Hospital visits from MIMIC III database	GAN	(-) Limited to generating discrete synthetic EHRs

### Medical text data

Medical texts are an important part of an EHR, reflecting the medical assessments and decisions. Reading these texts can put the underlying EHR at risk of identification, and therefore, privacy is regarded as an important objective when it comes to the SHR. This was confirmed by our study as privacy was the objective of the 9 studies out of the 12 studies that participated in this survey with various case studies. Table 3 lists the findings of the survey on the generative models for synthetic text data.

**Table 3:** The reviewed publications on generating synthetic medical records of text data

Paper & Year	Study Objective	Case Study & Language	Method	Key Takeaways
^21^, 2023	Privacy	English clinical notes from inpatient, outpatient, and emergency settings	GPT-3	(+) The physicians’ Turing test shows readability and clinical relevance of generated clinical text
^20^, 2023	Privacy	clinical case corpus in French (e.g., medical history of patients, treatment received at the hospital)	GPT-2, BLOOM	(-) Lack of the privacy leakage evaluation
^19^, 2023	Data scarcity	Emergency department notes in English (e.g., medical history, nurse’s observations)	GPT-2	(+) GPT-2 outperformed word swap and word-embedding techniques
^71^, 2023	Privacy, security	Medical description data in English from Centers for Medicare and Medicaid Services (https://www.cms.gov/) public healthcare records	Recurrent VAE	(+) Extending synthetic text data generator to the federated learning scenario
^72^, 2022	Privacy	Work-related injury description records in English, hospital visits from MIMIC III database	Perturbation-based data sifting	(+) Generating synthetic datasets with individual-level data obfuscation while maintaining population-level information
^73^, 2021	Data scarcity	English texts with temporal information of sleep-related activities	BERT	(+) Extracting temporal information from the user-generated text
^74^, 2021	Privacy	Laboratory messages in English pertaining to Salmonella	Sequence GAN	(-) Ineffective generating long laboratory messages
^75^, 2021	Privacy	Hospital reports in English from MIMIC III database	GPT-2	(+) Generating synthetic texts with the removed personal information and differential privacy
^76^, 2021	Privacy	Medical Dutch text reports from elderly care, mental care, and disabled care domains	LSTM, GPT-2	(+) LSTM produces synthetic text with higher precision compared to GPT-2, on the other hand, GPT-2 generates more coherent samples
^77^, 2019	Privacy, Class imbalance	Chinese EHR texts (e.g., personal information, history of present and past illnesses)	cGAN	—
^78^, 2019	Privacy	Text cancer pathology reports in English	Sequence GAN	(-) Lack the privacy leakage evaluation)
^79^, 2019	Class imbalance	Gender prediction based on user clicks on articles of a health-based website	Sequence GAN	(+) Sequence GAN outperforms the minority oversampling technique
^80^, 2018	Privacy	≈ 5.8M visit records (e.g., age, gender, discharge diagnosis code) in English	Encoder-Decoder LSTM	—

In contrast with the synthetic health time series generation where the proposed models were dominantly based on the GAN, GPT-style models are employed in approximately 40% of studies, surpassing individual usage rates of other generative methods. This is justifiable considering the versatility of GPT models. Such elaborative capabilities of GPT provided the ability to generate synthetic medical texts in different languages, spanning from far eastern countries, e.g., Chinese, to European countries, e.g., Dutch, and English (see the case studies in Table 3). Nevertheless, the GAN-based models were seen in some studies.

## Discussion

The need for a rich dataset for training ML methods on the one hand, and the difficulties in collecting patient data, e.g., privacy issues, on the other hand, make the generation of SHR a practical strategy. This is subjected to a high level of security in terms of re-identification along with acceptable fidelity. Countries adopt different regulations that intensively restrict the sharing of patient data, which is sometimes administered in a federated way. The use of SHR allows sharing of data, which can be employed by researchers to develop advanced ML methods for different medical applications, where access to the real data is problematic. Another application of SHR is the cases in which a heavy class imbalance negatively affects the learning process. In such cases, generative models are employed to create synthetic medical data for the minority classes. This is different from data augmentation where the minority data is reproduced, as the statistical distribution of the data is taken into account for generating SHRs.

Several surveys and review studies have been previously conducted on different models for generating SHRs. However, a comprehensive study on this topic with sufficient pervasiveness to explore different aspects of the studies, cannot be found in the existing literature, from a practical perspective. In addition, unlike other review papers (Table 4), ours covers more data modalities and DL models, thereby providing readers with novel perspectives on the topic. The outcomes of such a pervasive study can unveil practical limitations and bottlenecks of the existing methodologies to choose an appropriate model for such a demanding application.

**Table 4:** The reviewed publications on generating synthetic health records

Paper	Year	Data Modality			DL Model					Study Objective			
		Time-Series	Text	Longitudinal	GAN	LLM	Diff.	Auto-Encoder	Prob. Gen. Model	Data Imputation	Privacy	Class Imbalance	Data Scarcity
^10^	2023	✗	✗	✗	✗	*✓*	*✓*	✗	*✓*	*✓*	✗	*✓*	*✓*
^18^	2023	✗	*✓*	✗	*✓*	*✓*	✗	*✓*	*✓*	✗	*✓*	*✓*	✗
^31^	2022	✗	*✓*	*✓*	✗	*✓*	*✓*	✗	*✓*	*✓*	✗	*✓*	✗
^81^	2022	*✓*	✗	*✓*	✗	✗	*✓*	✗	*✓*	*✓*	✗	*✓*	✗
^82^	2022	*✓*	*✓*	*✓*	✗	*✓*	*✓*	✗	*✓*	*✓*	✗	✗	✗
^4^	2022	✗	*✓*	✗	✗	*✓*	*✓*	*✓*	*✓*	✗	✗	✗	✗
Ours	2023	✗	✗	✗	✗	✗	✗	✗	✗	✗	✗	✗	✗

Review publications on synthetic health data generation.

This study provided a scoping review of the most popular generative models for producing SHRs. Our analysis showed that the researchers employed GAN-based models most for generating synthetic time series compared to the other alternatives (See Supplementary Note 1). In addition to the GANs, the probabilistic models were widely used for generating synthetic longitudinal data. However, several studies reported that GAN-based models generally suffered from (i) the mode collapse issue^12–16^, (ii) requiring preliminary experiments to identify optimal hyperparameters, and (iii) having biases towards high-density classes^11^. Diffusion models demonstrated promising results in synthesizing time series compared to GANs. Nevertheless, resolving expensive computational costs and interoperability difficulties of diffusion models, are considered ongoing research endeavors. Finally, current works could gain significant advantages by integrating domain-specific expertise from physicians into the learning process^17,18^.

Generating synthetic clinical notes is a less explored area in the literature. Recent advancements of LLMs^19–21^ have demonstrated significant improvements in generating synthetic clinical notes. Regardless, LLMs suffer from requiring massive processing power (^21^ leveraged 560 A100 GPUs for 20 days to train the LLM). Furthermore^22,23^, addressed that LLMs struggle with complex reasoning problems. Non-reasoned outputs for generating synthetic clinical notes lacked coherence, consistency, and certainty^22^. Chain-of-Thought prompting^24^ standed out as a leading method aimed to improve complex reasoning capabilities through intermediate suggestions. While Chain-of-Thought has shown promising results, its effectiveness in tackling the reasonability of LLMs for complex multi-modal input and tasks necessitating compositional generalization remains an unresolved problem^22^. Despite the success of generative models, tweaking effort was sometimes required to achieve optimal performance^25^. It is worth mentioning that despite the success of studied papers in generating SHRs, the reproducibility of results of several studies is under question as (i) the implementation code is not available, and (ii) details of training hyper-parameters have not been reported.

Generating SHR necessitates real datasets to train the generative model, and the quality of the training data accessible defines the caliber of synthetic data^4,26–30^. The EHRs collected at healthcare sites are usually multi-dimensional and longitudinal datasets, recording patient history over multiple visits. However, secondary use of this data is restricted by privacy laws^31–33^; nevertheless, there are several de-identified datasets available for generating synthetic data. The popular databases for generating SHRs used by eligible publications of this review study can be found in Supplementary Table 1. Our findings show that despite the existence of public datasets available for generating SHRs, the majority of public longitudinal medical datasets primarily focus on ICU records, prioritizing acute patient cases and overlooking non-acute medical conditions. Furthermore, these public datasets often lack a comprehensive representation of all demographic groups and geographic regions, which limits their relevance and generalizability to broader populations. Finally, it is worth noting that most longitudinal records are reported in English, underscoring the current shortage of public resources across diverse languages.

**Fig. 3:**
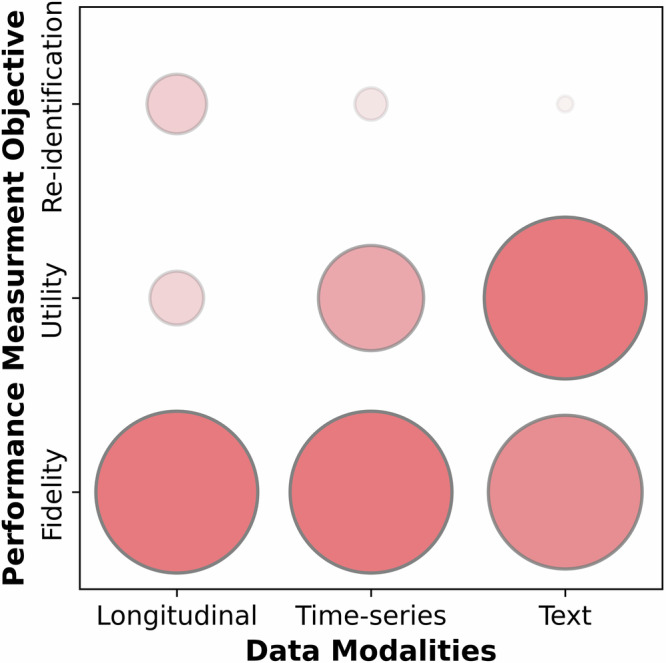
Normalized distribution of performance measurement objectives over the data modalities. Larger circles display more publications in each category. The Figure indicates an inadequate evaluation of re-identification of SHRs in studied papers. In addition, evaluating the utility of longitudinal data has been less researched compared to medical time series and text data.

One of the objectives of this survey was to help researchers find an optimal generative model for SHR among a great variety of existing ones, which in turn demanded a set of objective performance measures for comparison. Various statistical and intuitive metrics have been employed for comparing the performance of generative models which made choosing an optimal model for a case study complicated. In addition, some of the metrics are based on the discrimination power of the physicians^11,17,34,35^, whereas some others rely on the performance of a benchmark binary classifier to distinguish between SHR and EHR^8,36^. Supplementary Note 2 elaborates further on the techniques used for evaluating SHRs, while Supplementary Table 2 compiles the metrics that the included papers employed to assess the generated synthetic data. An important challenge, observed in this study is the lack of generic methods and metrics for comparing the performance of different generative models. Figure 3 represents the distribution of the three performance measurement objectives: fidelity, utility, and re-identification, over the data modalities. As can be seen, generating high-fidelity SHRs appears to be the main objective for all data modalities. It is implied from Fig. 3 that introducing appropriate performance measures to address the privacy of SHR is a contemporary research gap due to the shortage of pertinent studies. This is the case for the utility when it comes to the longitudinal SHR.

Statistical diversity of the data has been recently introduced as a measure for comparing the utility and fidelity of SHRs^28,37^. Furthermore, fairness of SHR was also defined as another comparative objective for the performance measures^38^. These two measures were not widely accepted by the researchers according to the citation records. All in all, there are no best-established systematic criteria or practices on how to evaluate SHRs.

Generative models for time series prediction showed promising results in various medical applications for classification and identification problems^39,40^. This topic was indeed an initiation for creating SHR, as was previously reported by reviewed studies. Recent scientific endeavors revealed that the application of SHR is not limited to the privacy of patient data, but can be extended towards statistical planning for clinical trials^41^, and evermore, towards addressing ethical issues^42^ by solving bias (e.g., black patients were less likely to be admitted to cardiology for heart failure care^43^) in the original dataset. In terms of methodology, the recent methodological trend of the practical models for the creation of SHR shows a shift from the GAN-based methods to the statistical models such as graph neural networks and diffusion models^9,11^, and data fairness was addressed by one of the recent studies^38^.

For further reading, we recommend the following key papers that complement our work and provide a deeper understanding of the subject of generating SHR.^27^ demonstrated that the evaluation metrics currently available for generic LLMs lack an understanding of medical and health-related concepts, which aligns closely with the findings of our study. In addition, the authors introduced a comprehensive collection of LLM-based metrics tailored for the evaluation of healthcare chat-bots from an end-user perspective. Social determinants of health (SDoH) encompass the conditions of individuals’ lives, influenced by the distribution of resources and power at various levels, and are estimated to contribute to 80–90% of modifiable factors affecting health outcomes^44^. However, documentation of SDoH is often incomplete in the structured data of EHRs. In^44^, the authors extracted six categories of SDoH from the EHR using LLMs to support research and clinical care. To address class imbalance and fine-tune the extraction model, the authors leveraged an LLM to generate synthetic SDoH data.

## Methods

### Definitions

The methodological contents of the reviewed papers addressed different study objectives, identified by their applicative terms. The main objectives of the introduced methods are summarised as follows:

- **Privacy:** Reliable SHRs can be generated based on patient data to be utilized for training and validation of ML methods.
- **Class Imbalance:** In many applications of health studies, access to different classes of data is not feasible in a consistent form, and a single class is dominantly seen in the study population. This can introduce bias to ML methods towards better learning of the dominant class. A reliable SHR can be invoked to generate synthetic data for minority classes.
- **Data Scarcity:** Access to data of a specific class can sometimes be problematic. In this case, the scarce class is identified and modeled using the absolute minority samples along with meta-learning methods. The SHR is generated based on the identified model to explore the characteristics of the scarce class.
- **Data Imputation:** EHR is heavily sparse with missing values, which are not uniformly obtained over the visits. Data imputation implies the methods to estimate the missing values that happened systematically or randomly in data collection.

### Generative models

The existing methods for generating synthetic data typically fall into two categories: probability distribution techniques and neural network-based methods^4^. Probability distribution techniques involve estimating a probability distribution of the real data and then drawing random samples that fit such distribution as synthetic data. Generative Markov-Bayesian probabilistic modeling is a technique used for synthesizing longitudinal EHRs^35^. On the other hand, Recent developments in synthetic data generation are adopting advanced neural networks. Below are the most commonly used neural network-driven generative models:

- **Generative adversarial networks (GANs)**^4^, comprising a generator and a discriminator, produce synthetic data resembling real samples drawn from a specific distribution. The discriminator distinguishes between real and synthetic samples, refining the generator’s ability to create realistic data through adversarial training, enabling accurate approximation of the data distribution and generation of high-fidelity novel samples. GANs can generate sequences of data points that closely resemble the patterns observed in the original image or time series data.
- **Diffusion models**^18^ gradually introduce noise into original data until it matches a predefined distribution. The core idea behind diffusion models is to learn the process of reversing this diffusion process, allowing for the generation of synthetic samples that closely resemble the original data while capturing its essential characteristics and variability.
- **Variational auto-encoders (VAEs)**^4^ are a category of generative models that learn to encode and decode data points while approximating a probability distribution, typically Gaussian, in the latent space. VAEs are trained by optimizing a variational lower bound on the log-likelihood of the data, enabling them to learn meaningful representations and create new data samples.
- **Large language models (LLMs)**^21^ can predict the probability of the next word in a text sequence based on preceding words, typically leveraging transformer architectures adept at capturing long-range dependencies. LLMs are effective for generating contextually appropriate text by learning the probability distribution of natural language data on vast corpora of text.

### Performance Measurement

Evaluating the strengths and weaknesses of generative models has become increasingly critical as these models continue to advance in complexity and capacity. The evaluation of generative models can be seen from different perspectives. In this study, we categorized evaluation metrics based on three different objectives: (i) **Fidelity:** degree of faithfulness in which the synthetic data preserves the essential characteristics, structures, and statistical properties of the actual data. Fidelity can be seen in either population-based (e.g., examining marginal and joint feature distributions) or individual-based (e.g., synthetic data must adhere to specific criteria, such as not including prostate cancer in a female patient) levels, (ii) **Re-identification:** concerning protection of sensitive information and confidentiality of individuals’ identity, and (iii) **Utility:** using synthetic data as a substitute for actual data for training/testing any medical devices and algorithms.

### Study Taxonomy

The study on SHRs covers a wide scope spanning from tabular medical data of a single record to longitudinal data from several visits and events. In contrast with the varieties in the adoption of different learning methods to create synthetic health data, the methodological suitability of the proposed methods depends merely on data modality. This review study is hence, performed according to the following taxonomy: (i) longitudinal medical/health data, (ii) medical/health time series, and (iii) medical/health texts.

### Research method

To perform this scoping review, we followed the recommendations outlined in the PRISMA-ScR guidelines^45^. Supplementary Note 3 provides the PRISMA-ScR checklist. The research method was comprised of 5 steps, described as follows:

1. **Search:** A systematic search is performed on the three widely accepted platforms of scientific publications in this domain: PubMed, Web of Science, and Scopus using combinations of {Synthetic}, {Time Series, Text, Longitudinal}, and {Medical, Medicine, Health} keywords in the title and/or abstract of the publications. Our search queries are shown in Fig. 1b. We adapted the string for each database, using various forms of the terms.
2. **Identification:** The outcomes of the search were explored in terms of duplication and repeated publications were excluded from the study.
3. **Screening:** In this phase Title and Abstract of the identified papers were studied and the topical relevance of the publications was investigated. Some of the publications from a different scientific topic were identified to participate in the study because of similarities in keywords. These publications were detected and excluded from the study.
4. **Eligibility (inclusion criteria):** After the search phase, only those publications fulfilling all the below criteria were allowed to participate in the study: (i) published within 2018–2023, (ii) the full paper is available, and (iii) addressing an ML topic for electronic health record (EHR) generation. Papers with only the Abstract available, cannot be analyzed and hence, excluded from the study, in addition to those addressing synthetic organs, without addressing the ML objectives.
5. **Included Studies:** This study focuses on reproducible ML methods for generating synthetic, time series, longitudinal, or text contents of medical record. Therefore, the validity of the proposed methods in terms of implementation feasibility is an important criterion for consideration. We consolidated the scientific quality of the study by using the following Exclusion Criteria: (i) lack of the peer-reviewed process for the publication, (ii) EHR generation is not the major objective of the publication, and (iii) limited to tabular and image data only.

## Supplementary information

Supplementary Material

## Data Availability

The raw data employed for this study was entirely obtained from the public datasets using three well-known search engines: PubMed, Scopus, and Web of Science. The research methodology is fully described in the text, where inclusion and exclusion criteria are completely described.

## Code availability

Not applicable. No codes were developed for the analysis.
