# Supplementary material for: A review on generative AI models for synthetic medical text, time series, and longitudinal data
Source: NPJ Digit Med. 2025 May 15;8:281. doi: 10.1038/s41746-024-01409-w (PMC12081667; doi:10.1038/s41746-024-01409-w)
Supplement: Supplementary file 1 — Supplementary Material [file 41746_2024_1409_MOESM1_ESM.pdf]

Supplementary Information

A Review on Generative AI Models for Synthetic Medical Text, Time Series, and Longitudinal Data

Mohammad Loni[1], Fatemeh Poursalim[2], Mehdi Asadi[3], Arash Gharehbaghi[4,*]
[1]School of Innovation, Design and Engineering, Mälardalen University, Västerås, Sweden
[2]Servicehälsan Familjeläkare i Västerås AB, Västerås, Sweden
[3]ICT Department, Turku University of Applied Sciences, Turku, Finland
[4]Department of Biomedical Engineering, Linköping University, Linköping, Sweden

[*] Corresponding Author: Arash Gharehbaghi, Email: arash.gharehbaghi@liu.se

Contents

Supplementary Note 1. Structured Abstract 3

Supplementary Table 1. Summary of Public Datasets for Synthetic Data Generation 4

Supplementary Note 2. SHR Evaluation Techniques 9

Supplementary Table 2. A Summary of the Metrics Used to Evaluate Generative Models for Creating Synthetic Health Records 10

Supplementary Note 3. PRISMA-ScR Checklist 12

Supplementary References 16

Supplementary Note 1. Structured Abstract

Background: Generating synthetic health records has recently become a research topic. A database of synthetic health records (SHRs), as a copy of electronic health records, can be used for training machine learning (ML) methods and educational purposes. This approach eliminates the need to use the real patient data, thereby ensuring the preservation of data privacy. Electronic health records (EHRs) can contain tabular data, longitudinal data, time series, images, and texts.

Objectives: 1) Finding state-of-the-art generative models for creating synthetic medical texts, time series, and longitudinal data, along with the methodological limitations. 2) Summarizing the existing performance measures in conjunction with the related metrics for evaluating the quality of SHR. 3) Listing the most used datasets employed by the researchers for generating SHR. 4) Finding the key research gaps of the field.

Eligibility criteria: 1) Published studies within 2018–2023. 2) Full paper is available. 3) Addressing an ML topic for electronic health record generation. 4) The papers describing synthetic organs, without addressing the ML objectives were excluded.

Sources of evidence: A systematic search is performed on the three widely accepted platforms of scientific publications in this domain: PubMed, Web of Science, and Scopus.

Charting method: Tabular and graphical representations.

Results: 52 publications fulfilled the inclusion and exclusion criteria and ultimately participated in the study (PubMed=27, Scopus=19, and Web of Science=6). Generation of synthetic physiological time series was observed in 22 reports (42%) of the published peer-reviewed papers, from which synthetic Electrocardiogram is the most common case study (10 studies). Electroencephalogram was the main topic of the second most common objective where the diffusion model resulted in the optimal utility. Privacy is the main objective of 16 out of the 17 studies with various case studies comprising kidney diseases, patients with hearing loss, Parkinson's and Alzheimer's diseases, chronic heart failure disease, diabetes, hypertension, and hospital admissions. As for the medical texts, 9 studies out of the 12 studies that participated in this survey with various case studies.

Conclusion: 1) GAN was seen to be the dominant model for the two data modalities, time series and longitudinal data, where fidelity is the main objective of the performance measurement for both cases. 2) LLM received the most popularity for generating synthetic medical text, where the utility was found to be the major performance measurement explored as the study objective. 3) Privacy was observed to be the dominant objective of creating SHR, even though other objectives such as class imbalance and data scarcity were widely studied. 4) The development of the appropriate evaluation metrics is considered a major research gap.

Supplementary Table 1. Summary of public datasets for synthetic data generation

Dataset	URL	Case Study	Data Modality: Time Series	Data Modality: Text	Data Modality: Longitud.	Included Study Ref.
MIMIC III [1]	Link	De-identified patient records admitted to intensive care unit (ICU) from 2001 to 2012 containing demographics, laboratory test results, procedures, caregiver notes, medications, imaging reports, mortality, etc.	✔	✔	✔	[2–11]
MIMIC IV [12]	Link	Critical care data for patients admitted to the ICU at the Beth Israel Deaconess Medical Center. The information available includes patient measurements, orders, diagnoses, procedures, treatments, and clinical notes	✔	✔	✔	[13]
eICU [14]	Link	A critical care database spanning multiple centers holds information from over 200,000 admissions to ICUs from 208 hospitals situated across the U.S.	✔	✔	✔	[11, 15, 16]
HiRID [17]	Link	a high-resolution ICU dataset relating to more than 3 billion observations from ≈34,000 ICU patient admissions	✔		✔	[11]
Pile [18]	Link	An 825 GiB English text corpus from 22 diverse high-quality subsets including the medical domain		✔		[19]

Continued on the next page...

Table4 – Continued from previous page...

Dataset	URL	Case Study	Data Modality			Included Study Ref.
			Time Series	Text	Longitud.	
E3C [20]	Link	A multilingual (English, French, Italian, Spanish, and Basque) corpus database containing biomedical documents extracted from different sources, including journals, existing biomedical corpora, etc.		✔		[21]
INPCR [22]	Link	This database stands as one of the most extensive health information exchange in U.S., encompassing more than 100 distinct healthcare organizations contributing data. The database contains information on over 18 million patients, comprising 10 billion clinical observations, and over 147 million text reports.		✔		[23, 24]
Administrative Health Records [25]	Link	Patients received a prescription for an opioid during the 7-year study window. Data includes demographic information, laboratory tests, prescription history, hospitalizations, etc.			✔	[26]
PPMI [27]	Link	Observational clinical study containing 354 Parkinson patients who participated in a range of clinical, neurological, and demographic assessments			✔	[28]
NACC [29]	Link	Storing patient-level Alzheimer's disease data collected from 2284 patients across multiple clinics			✔	[28]

Continued on the next page...

Table4 – Continued from previous page...

Dataset	URL	Case Study	Data Modality			Included Study Ref.
			Time Series	Text	Longitud.	
Evotion [30]	Link	Information relating to patterns of real-world hearing aid usage and sound environment exposure. EVOTION contains longitudinally observations from 53 individuals and includes the following measures: the sound environment, the hearing aid setting, timestamps, ID, and the degree of hearing loss on the best hearing ear of the individuals			✔	[31]
SEER [32]	Link	This database provides information on cancer incidence and survival from population-based cancer registries in 22 U.S. geographic areas			✔	[33]
Human Activity Sensing Archive [34]	Link	A large-scale human activity corpus archive	✔			[35, 36]
UCR Time Series Archive [37]	Link	A large repository of time series datasets including health records of Electrocardiogram (ECG), motion, etc.	✔			[38]
Autonomic Aging [39]	Link	A database of high-resolution biological signals to describe the effect of healthy aging on cardiovascular regulation	✔			[40]
PTB-XL [41]	Link	A large dataset of 21799 clinical 12-lead ECGs from 18869 patients. The raw waveform data was annotated by up to two cardiologists	✔			[42]
AF Classification Challenge [43]	Link	A dataset of single-lead ECGs (between 30 s and 60 s in length) with normal sinus rhythm, atrial fibrillation (AF), an alternative rhythm, or noisy classes	✔			[44]

Continued on the next page...

Table4 – Continued from previous page...

Dataset	URL	Case Study	Data Modality			Included Study Ref.
			Time Series	Text	Longitud.	
UniMiB [45]	Link	A dataset tailored for human activity recognition and fall detection with 11,771 acceleration samples performed by 30 subjects aged between 18 and 60 years	✔			[46]
PAMAP2 [47]	Link	The PAMAP2 Physical Activity Monitoring dataset contains data of 18 different physical activities (e.g. walking, cycling, playing soccer), performed by 9 subjects wearing 3 inertial measurement units and a heart rate monitor	✔			[35]
MIT-BIH Arrhythmia [48]	Link	This dataset contains 48 30-min excerpts of two-channel ECG recordings, obtained from 47 subjects studied between 1975 and 1979	✔			[49–51]
MIT-BIH Normal Sinus Rhythm (NSR) [48]	Link	This dataset includes 18 long-term ECG recordings of subjects with no significant arrhythmias	✔			[49]
Sleep-EDF (Expanded) [52]	Link	This dataset contains 197 whole-night PolySomnoGraphic sleep recordings, containing EEG, Electrooculography, chin electromyography, and event markers. Some records also contain respiration and body temperature	✔			[53]
The National Sleep Research Resource [54]	Link	Compilation of annotated sleep datasets, along with interfaces and tools for accessing and analyzing this data, is available	✔			[55]

Continued on the next page...

Table4 – Continued from previous page...

Dataset	URL	Case Study	Data Modality			Included Study Ref.
			Time Series	Text	Longitud.	
UCI EEG Dataset	Link	This dataset examines EEG correlates of genetic predisposition to alcoholism. It contains measurements from 64 electrodes placed on the scalp sampled at 256 Hz	✔			[50]
PhysioNet Challenge 2015 [56]	Link	This database includes ECG and Photoplethysmogram (PPG) recordings from 750 patients that suffered either of the following cardiac conditions; asystole, extreme bradycardia, extreme tachycardia, ventricular tachycardia, and ventricular flutter	✔			[57]
PPG-DB [58]	Link	A collection of de-identified photoplethysmography data for studying cardiovascular disease. The dataset contains 657 records from 219 subjects, spanning ages 20 to 89 years, with records of diseases like hypertension and diabetes	✔			[57]
UCI ML Repository [59]	Link	A collection of databases in various subjects including health and medicine	✔	✔	✔	[33, 38, 60]
PhysioNet [48]	Link	An extensive collection of health data from both healthy individuals and those dealing with conditions like sudden cardiac death, congestive heart failure, epilepsy, gait disorders, sleep apnea, and aging	✔	✔	✔	[46, 61]

Supplementary Note 2. SHR Evaluation Techniques

Evaluation techniques can also be categorized into two main groups: quantitative and qualitative methods: (i) **Quantitative evaluation Methods:** Supplementary Table 2 represents different quantitative evaluation metrics along with the evaluation objectives used to assess the effectiveness of generative models for the reviewed publications. (ii) **Qualitative evaluation Methods:** Qualitative evaluation methods are often employed alongside quantitative measures to complement results with straightforward assessments. For instance, many studies utilize visualization approaches to compare distributions and embeddings of synthetic and real data, such as using histogram [31,44], Q–Q-plot [44], t-SNE [7,38,46], PCA [38,46], and correlation [6,31]. Concerns regarding the clinical validity and trustworthiness of synthetic data pose significant obstacles to using it for clinical research. To tackle this issue, some studies have performed clinician evaluations, wherein medical professionals evaluate the realism of the synthetic data they are presented with [19,42,44,55,62].

Supplementary Table 2. A Summary of the Metrics Used to Evaluate Generative Models for Creating Synthetic Health Records

Objective	Method	Data Modality			Included Study Ref.
		Time Series	Text	Longitud.	
Fidelity	Discriminative score [63]	✔		✔	[11, 35]
	Maximum mean discrepancy (MMD) [64]	✔		✔	[11, 38, 51, 57, 61, 65]
	Multivariate Hellinger distance [66]			✔	[26]
	Wasserstein distance [64]	✔		✔	[67–70]
	Inception score (IS) [63]	✔		✔	[16, 53]
	Wilcoxon rank sum test [71]	✔			[50]
	Kolmogorov–Smirnov (K-S) test [63]	✔		✔	[6, 8, 44, 67, 68]
	Euclidean distance (ED) [71]	✔		✔	[16, 26, 35, 72]
	Dimension-wise probability (DWPro) [73]			✔	[6, 11, 33]
	Dimension-wise prediction (DWPre) [73]			✔	[8, 33]
	Pairwise distance correlation [71]	✔			[67]
	Pearson correlation [74]		✔	✔	[11, 19, 69]
	P-Value test	✔	✔	✔	[5, 50, 75, 76]
	Spearman rank correlation [74]			✔	[28, 69]
	Kendall's rank correlation [74]			✔	[69]
	Kullback–Leibler (KL) -divergence [63]	✔		✔	[28, 53, 77]
	Jensen–Shannon (JS) -divergence/-distance [78]	✔		✔	[28, 46, 67, 70, 79]
	Cosine similarity [80]	✔	✔		[9, 46]
	Weighted latent difference [63]			✔	[77]
	Bilingual evaluation understudy (BLEU), self-BLEU [81]		✔		[9, 10, 21, 23, 24, 82, 83]
	Jaccard similarity [84]		✔		[10]
	G^2-test [85]		✔		[10]

Continued on the next page...

Table 4 – Continued from the previous page...

Objective	Method	Data Modality: Time Series	Text	Longitud.	Included Study Ref.
	NLL-test [86] (likelihood-based)		✔		[10, 82]
	Recall-oriented understudy for gisting evaluation (ROUGE) [87]		✔		[83, 88]
	N-grams overlap score [21]		✔		[21]
	Consensus-based image description evaluation (CIDEr) [89]		✔		[83]
	Exact match difference [89]		✔		[76]
Re-identification	Membership inference attack (MIA) [63]	✔		✔	[3, 4, 11, 51, 65, 77]
	Attribute disclosure attack [63]			✔	[4, 6, 77, 90]
	Differential privacy [63]	✔	✔	✔	[6, 7, 11, 16]
	Keywords inference attack		✔		[91]
	Bayesian disclosure attack [92]			✔	[5]
Utility	Sensitivity, specificity [93]	✔	✔		[23, 24, 50]
	Precision [93]	✔	✔		[19, 38, 88, 94]
	Recall [93]	✔	✔	✔	[4, 19, 38, 88, 94]
	F1-score [93]	✔	✔		[3, 19, 23, 24, 38, 76, 83, 88, 94, 95]
	Area under the receiver operating characteristic curve (AUROC) [93]	✔	✔	✔	[2–4, 23, 24, 33, 42, 44, 57, 70, 75, 77, 79, 90, 95]
	Predictive score [96]	✔			[35]
	Longitudinal imputation perplexity (LPL) and cross-modality imputation perplexity (MPL) [4]			✔	[4]
	Dynamic time warping (DTW) [97]	✔			[35, 51]
	Multivariate dynamic time warping (MVDTW) [97]	✔			[65, 72]

Supplementary Note 3. Systematic Reviews and Meta-Analyses Extension for Scoping Reviews (PRISMA-ScR) Checklist

SECTION	ITEM	PRISMA-ScR CHECKLIST ITEM	Location in the paper	Remarks
**TITLE**				
Title	1	Identify the report as a scoping review. Click here to enter text.	Page 1	-
**ABSTRACT**				
Structured summary	2	Provide a structured summary that includes (as applicable): background, objectives, eligibility criteria, sources of evidence, charting methods, results, and conclusions related to the review questions and objectives.	Supplementary Note 1	-
**INTRODUCTION**				
Rationale	3	Describe the rationale for the review in the context of what is already known. Explain why the review questions/objectives lend themselves to a scoping review approach.	Pages 1-2	-
Objectives	4	Provide an explicit statement of the questions and objectives being addressed with reference to their key elements (e.g., population or participants, concepts, and context) or other relevant key elements used to conceptualize the review questions and/or objectives.	Page 2	-
**METHODS**				
Protocol and registration	5	Indicate whether a review protocol exists; state if and where it can be accessed (e.g., a Web address); and if available, provide registration information, including the registration number.	N/A	*The online registration is not available.*
Eligibility criteria	6	Specify characteristics of the sources of evidence used as eligibility criteria (e.g., years considered, language, and publication status), and provide a rationale.	Page 7	-

Continued on the next page...

SECTION	ITEM	PRISMA-ScR CHECKLIST ITEM	Location in the paper	
Information sources*	7	Describe all information sources in the search (e.g., databases with dates of coverage and contact with authors to identify additional sources), as well as the date the most recent search was executed.	Page 7	-
Search	8	Present the full electronic search strategy for at least 1 database, including any limits used, such that it could be repeated.	Figure 1	-
Selection of sources of evidence†	9	State the process for selecting sources of evidence (i.e., screening and eligibility) included in the scoping review.	Page 7	-
Data charting process‡	10	Describe the methods of charting data from the included sources of evidence (e.g., calibrated forms or forms that have been tested by the team before their use, and whether data charting was done independently or in duplicate) and any processes for obtaining and confirming data from investigators.	N/A	-
Data items	11	List and define all variables for which data were sought and any assumptions and simplifications made.	N/A	-
Critical appraisal of individual sources of evidence§	12	If done, provide a rationale for conducting a critical appraisal of included sources of evidence; describe the methods used and how this information was used in any data synthesis (if appropriate).	N/A	-

Continued on the next page...

SECTION	ITEM	PRISMA-ScR CHECKLIST ITEM	Location in the paper	
Synthesis of results	13	Describe the methods of handling and summarizing the data that were charted.	Pages 2 to 5	*The results were represented in new graphical illustrations in which the processing methods, research objectives, and data modalities found in the review were demonstrated (Figures 2–3). Moreover, new tabular representations listed the findings and related the state-of-the-art to the applications for the three data modalities, medical time series, longitudinal record, and texts, independently.*

RESULTS

Selection of sources of evidence	14	Give numbers of sources of evidence screened, assessed for eligibility, and included in the review, with reasons for exclusions at each stage, ideally using a flow diagram.	Page 3 and Figure 1	-
Characteristics of sources of evidence	15	For each source of evidence, present characteristics for which data were charted and provide the citations.	Pages 3–6	*Table 1 listed the evidence of the findings for medical time series. The evidence for the medical longitudinal records and text were included in Table 2 and Table 3, respectively.*
Critical appraisal within sources of evidence	16	If done, present data on critical appraisal of included sources of evidence (see item 12).	N/A	
Results of individual sources of evidence	17	For each included source of evidence, present the relevant data that were charted that relate to the review questions and objectives.	Pages 3–6	*Medical time series is a group of medical records where the synthetic data found its importance, as addressed by the study objectives. Synthetic ECG is a typical example of such an application. Medical longitudinal data was found to be the most important part of the EHR. Creating a synthetic EHR, named SHR, is an important research question. Synthetic medical text is another topic that has been newly added to this topic. This was well in accordance with the study objectives.*

Continued on the next page...

SECTION	ITEM	PRISMA-ScR CHECKLIST ITEM	Location in the paper	
Synthesis of results	18	Summarize and/or present the charting results as they relate to the review questions and objectives.	Pages 3-4	*Sections Results and Discussions (Table 1, Table 2, Table 3, and Table 4)*
**DISCUSSION**				
Summary of evidence	19	Summarize the main results (including an overview of concepts, themes, and types of evidence available), link to the review questions and objectives, and consider the relevance to key groups.	Pages 3-4	-
Limitations	20	Discuss the limitations of the scoping review process.	Page 2 and Page 7	*The scoping review didn't consider synthetic medical images and tabular records. These two topics were already included in the existing publications*
Conclusions	21	Provide a general interpretation of the results with respect to the review questions and objectives, as well as potential implications and/or next steps.	Pages 4-5	-
**FUNDING**				
Funding	22	Describe sources of funding for the included sources of evidence, as well as sources of funding for the scoping review. Describe the role of the funders of the scoping review.	Page 7	-

Supplementary References

[1] Johnson, A. E. *et al.* Mimic-iii, a freely accessible critical care database. *Scientific data* **3**, 1–9 (2016).

[2] Bing, S., Dittadi, A., Bauer, S. & Schwab, P. Conditional generation of medical time series for extrapolation to underrepresented populations. *PLOS Digital Health* **1**, e0000074 (2022).

[3] Theodorou, B., Xiao, C. & Sun, J. Synthesize high-dimensional longitudinal electronic health records via hierarchical autoregressive language model. *Nature Communications* **14**, 5305 (2023).

[4] Wang, Z. & Sun, J. Promptehr: Conditional electronic healthcare records generation with prompt learning. *Conference on Empirical Methods in Natural Language Processing* 2873–2885 (2022).

[5] Zhou, N., Wang, L., Marino, S., Zhao, Y. & Dinov, I. D. Datasifter ii: Partially synthetic data sharing of sensitive information containing time-varying correlated observations. *Journal of Algorithms & Computational Technology* **16**, 17483026211065379 (2022).

[6] Kaur, D. *et al.* Application of bayesian networks to generate synthetic health data. *Journal of the American Medical Informatics Association* **28**, 801–811 (2021).

[7] Lee, D. *et al.* Generating sequential electronic health records using dual adversarial autoencoder. *Journal of the American Medical Informatics Association* **27**, 1411–1419 (2020).

[8] Baowaly, M. K., Lin, C.-C., Liu, C.-L. & Chen, K.-T. Synthesizing electronic health records using improved generative adversarial networks. *Journal of the American Medical Informatics Association* **26**, 228–241 (2019).

[9] Zhou, N., Wu, Q., Wu, Z., Marino, S. & Dinov, I. D. Datasiftertext: Partially synthetic text generation for sensitive clinical notes. *Journal of Medical Systems* **46**, 96 (2022).

[10] Al Aziz, M. M. *et al.* Differentially private medical texts generation using generative neural networks. *ACM Transactions on Computing for Healthcare* **3**, 1–27 (2021).

[11] Li, J., Cairns, B. J., Li, J. & Zhu, T. Generating synthetic mixed-type longitudinal electronic health records for artificial intelligent applications. *NPJ Digital Medicine* **6**, 98 (2023).

[12] Johnson, A. E. *et al.* Mimic-iv, a freely accessible electronic health record dataset. *Scientific data* **10**, 1 (2023).

[13] Nikolentzos, G., Vazirgiannis, M., Xypolopoulos, C., Lingman, M. & Brandt, E. G. Synthetic electronic health records generated with variational graph autoencoders. *NPJ Digital Medicine* **6**, 83 (2023).

[14] Pollard, T. J. *et al.* The eicu collaborative research database, a freely available multi-center database for critical care research. *Scientific data* **5**, 1–13 (2018).

[15] Lee, M., Tae, D., Choi, J. H., Jung, H.-Y. & Seok, J. Improved recurrent generative adversarial networks with regularization techniques and a controllable framework. *Information Sciences* **538**, 428–443 (2020).

[16] Wang, S., Rudolph, C., Nepal, S., Grobler, M. & Chen, S. Part-gan: Privacy-preserving time-series sharing. In *Artificial Neural Networks and Machine Learning*, 578–593 (Springer, 2020).

[17] Yèche, H. *et al.* Hirid-icu-benchmark–a comprehensive machine learning benchmark on high-resolution icu data. *arXiv preprint arXiv:2111.08536* (2021).

[18] Gao, L. *et al.* The pile: An 800gb dataset of diverse text for language modeling. *arXiv preprint arXiv:2101.00027* (2020).

[19] Peng, C. *et al.* A study of generative large language model for medical research and healthcare. *NPJ Digital Medicine* **6** (2023).

[20] Zanoli, R., Lavelli, A., do Amarante, D. V. & Toti, D. Assessment of the e3c corpus for the recognition of disorders in clinical texts. *Natural Language Engineering* 1–19 (2023).

[21] Hiebel, N., Ferret, O., Fort, K. & Névéol, A. Can synthetic text help clinical named entity recognition? a study of electronic health records in french. In *17th Conference of the European Chapter of Association for Computational Linguistics* (2023).

[22] McDonald, C. J. *et al.* The indiana network for patient care: a working local health information infrastructure. *Health affairs* **24**, 1214–1220 (2005).

[23] Kasthurirathne, S. N., Dexter, G. & Grannis, S. J. Generative adversarial networks for creating synthetic free-text medical data: a proposal for collaborative research and re-use of machine learning models. *AMIA Summits on Translational Science Proceedings* **2021**, 335 (2021).

[24] Kasthurirathne, S. N., Dexter, G. & Grannis, S. J. An adversorial approach to enable re-use of machine learning models and collaborative research efforts using synthetic unstructured free-text medical data. *MEDINFO 2019: Health and Wellbeing e-Networks for All* 1510–1511 (2019).

[25] Sharma, V. *et al.* Characterisation of concurrent use of prescription opioids and benzodiazepine/z-drugs in alberta, canada: a population-based study. *BMJ open* **9**, e030858 (2019).

[26] Mosquera, L. *et al.* A method for generating synthetic longitudinal health data. *BMC Medical Research Methodology* **23**, 1–21 (2023).

[27] Marek, K. *et al.* The parkinson progression marker initiative (ppmi). *Progress in neurobiology* **95**, 629–635 (2011).

[28] Wendland, P. *et al.* Generation of realistic synthetic data using multimodal neural ordinary differential equations. *NPJ Digital Medicine* **5**, 122 (2022).

[29] Beekly, D. L. *et al.* The national alzheimer's coordinating center (nacc) database: the uniform data set. *Alzheimer Disease & Associated Disorders* **21**, 249–258 (2007).

[30] Christensen, J. H. *et al.* Fully synthetic longitudinal real-world data from hearing aid wearers for public health policy modeling. *Frontiers in Neuroscience* **13**, 850 (2019).

[31] Christensen, J. H. *et al.* Fully synthetic longitudinal real-world data from hearing aid wearers for public health policy modeling. *Frontiers in Neuroscience* **13**, 850 (2019).

[32] Hankey, B. F., Ries, L. A. & Edwards, B. K. The surveillance, epidemiology, and end results program: a national resource. *Cancer Epidemiology Biomarkers & Prevention* **8**, 1117–1121 (1999). URL https://seer.cancer.gov/.

[33] Li, R. *et al.* Improving an electronic health record–based clinical prediction model under label deficiency: Network-based generative adversarial semisupervised approach. *JMIR Medical Informatics* **11**, e47862 (2023).

[34] Kawaguchi, N. *et al.* Hasc challenge: gathering large scale human activity corpus for the real-world activity understandings. In *Proceedings of the 2nd augmented human international conference*, 1–5 (2011).

[35] Wang, J., Chen, Y. & Gu, Y. A wearable-har oriented sensory data generation method based on spatio-temporal reinforced conditional gans. *Neurocomputing* **493**, 548–567 (2022).

[36] Li, X., Luo, J. & Younes, R. Activitygan: Generative adversarial networks for data augmentation in sensor-based human activity recognition. In *ACM International Joint Conference on Pervasive and Ubiquitous Computing and Proceedings of the 2020 ACM International Symposium on Wearable Computers*, 249–254 (2020).

[37] Dau, H. A. *et al.* The ucr time series archive. *IEEE/CAA Journal of Automatica Sinica* **6**, 1293–1305 (2019).

[38] Yang, Z., Li, Y. & Zhou, G. Ts-gan: Time-series gan for sensor-based health data augmentation. *ACM Transactions on Computing for Healthcare* **4**, 1–21 (2023).

[39] Schumann, A. & Bär, K.-J. Autonomic aging–a dataset to quantify changes of cardiovascular autonomic function during healthy aging. *Scientific Data* **9**, 95 (2022).

[40] Festag, S. & Spreckelsen, C. Medical multivariate time series imputation and forecasting based on a recurrent conditional wasserstein gan and attention. *Journal of Biomedical Informatics* **139**, 104320 (2023).

[41] Wagner, P. *et al.* Ptb-xl, a large publicly available electrocardiography dataset. *Scientific data* **7**, 1–15 (2020).

[42] Alcaraz, J. M. L. & Strodthoff, N. Diffusion-based conditional ecg generation with structured state space models. *Computers in Biology and Medicine* 107115 (2023).

[43] Clifford, G. D. *et al.* Af classification from a short single lead ecg recording: The physionet/computing in cardiology challenge 2017. In *2017 Computing in Cardiology (CinC)*, 1–4 (IEEE, 2017).

[44] Asadi, M. *et al.* Accurate detection of paroxysmal atrial fibrillation with certified-gan and neural architecture search. *Scientific Reports* **13**, 11378 (2023).

[45] Micucci, D., Mobilio, M. & Napoletano, P. Unimib shar: A dataset for human activity recognition using acceleration data from smartphones. *Applied Sciences* **7**, 1101 (2017).

[46] Li, X., Metsis, V., Wang, H. & Ngu, A. H. H. Tts-gan: A transformer-based time-series generative adversarial network. In *International Conference on Artificial Intelligence in Medicine*, 133–143 (Springer, 2022).

[47] Reiss, A. & Stricker, D. Introducing a new benchmarked dataset for activity monitoring. In *2012 16th international symposium on wearable computers*, 108–109 (IEEE, 2012).

[48] Goldberger, A. L. *et al.* Physiobank, physiotoolkit, and physionet: components of a new research resource for complex physiologic signals. *circulation* **101**, e215–e220 (2000).

[49] Habiba, M., Borphy, E., Pearlmutter, B. A. & Ward, T. Ecg synthesis with neural ode and gan models. In *International Conference on Electrical, Computer and Energy Technologies*, 1–6 (IEEE, 2021).

[50] Maweu, B. M., Shamsuddin, R., Dakshit, S. & Prabhakaran, B. Generating healthcare time series data for improving diagnostic accuracy of deep neural networks. *IEEE Transactions on Instrumentation and Measurement* **70**, 1–15 (2021).

[51] Brophy, E. Synthesis of dependent multichannel ecg using generative adversarial networks. In *Proceedings of the 29th ACM international conference on Information & Knowledge Management*, 3229–3232 (2020).

[52] Kemp, B., Zwinderman, A. H., Tuk, B., Kamphuisen, H. A. & Oberye, J. J. Analysis of a sleep-dependent neuronal feedback loop: the slow-wave microcontinuity of the eeg. *IEEE Transactions on Biomedical Engineering* **47**, 1185–1194 (2000).

[53] Lee, W., Lee, J. & Kim, Y. Contextual imputation with missing sequence of eeg signals using generative adversarial networks. *IEEE Access* **9**, 151753–151765 (2021).

[54] Zhang, G.-Q. *et al.* The national sleep research resource: towards a sleep data commons. *Journal of the American Medical Informatics Association* **25**, 1351–1358 (2018).

[55] Wickramaratne, S. D. & Parekh, A. Sleepsim: Conditional gan-based non-rem sleep eeg signal generator. In *45th Annual International Conference of the IEEE Engineering in Medicine & Biology Society*, 1–4 (IEEE, 2023).

[56] Clifford, G. D. *et al.* The physionet/computing in cardiology challenge 2015: reducing false arrhythmia alarms in the icu. In *2015 Computing in Cardiology Conference (CinC)*, 273–276 (IEEE, 2015).

[57] Kiyasseh, D. *et al.* Plethaugment: Gan-based ppg augmentation for medical diagnosis in low-resource settings. *IEEE journal of biomedical and health informatics* **24**, 3226–3235 (2020).

[58] Liang, Y., Chen, Z., Liu, G. & Elgendi, M. A new, short-recorded photoplethysmogram dataset for blood pressure monitoring in china. *Scientific data* **5**, 1–7 (2018).

[59] Asuncion, A., Newman, D. *et al.* Uci machine learning repository (2007). URL https://archive.ics.uci.edu/.

[60] Boukhennoufa, I. *et al.* A novel model to generate heterogeneous and realistic time-series data for post-stroke rehabilitation assessment. *IEEE Transactions on Neural Systems and Rehabilitation Engineering* (2023).

[61] Nikolaidis, K. *et al.* Augmenting physiological time series data: A case study for sleep apnea detection. In *Joint European Conference on Machine Learning and Knowledge Discovery in Databases*, 376–399 (Springer, 2019).

[62] Wang, X. *et al.* Using an optimized generative model to infer the progression of complications in type 2 diabetes patients. *BMC Medical Informatics and Decision Making* **22**, 1–9 (2022).

[63] Ghosheh, G. O., Li, J. & Zhu, T. A survey of generative adversarial networks for synthesizing structured electronic health records. *ACM Computing Surveys* **56**, 1–34 (2024).

[64] El Emam, K., Mosquera, L., Fang, X. & El-Hussuna, A. Utility metrics for evaluating synthetic health data generation methods: validation study. *JMIR Medical Informatics* **10**, e35734 (2022).

[65] Brophy, E., De Vos, M., Boylan, G. & Ward, T. Multivariate generative adversarial networks and their loss functions for synthesis of multichannel ecgs. *Ieee Access* **9**, 158936–158945 (2021).

[66] Le Cam, L. M. & Yang, G. L. *Asymptotics in statistics: some basic concepts* (Springer Science & Business Media, 2000).

[67] Haleem, M. S. *et al.* Deep-learning-driven techniques for real-time multimodal health and physical data synthesis. *Electronics* **12**, 1989 (2023).

[68] Foomani, F. H. *et al.* Synthesizing time-series wound prognosis factors from electronic medical records using generative adversarial networks. *Journal of Biomedical Informatics* **125**, 103972 (2022).

[69] Shi, J., Wang, D., Tesei, G. & Norgeot, B. Generating high-fidelity privacy-conscious synthetic patient data for causal effect estimation with multiple treatments. *Frontiers in Artificial Intelligence* **5**, 918813 (2022).

[70] Yoon, J., Drumright, L. N. & Van Der Schaar, M. Anonymization through data synthesis using generative adversarial networks (ads-gan). *IEEE Journal of Biomedical and Health Informatics* **24**, 2378–2388 (2020).

[71] Murtaza, H. *et al.* Synthetic data generation: State of the art in health care domain. *Computer Science Review* **48**, 100546 (2023).

[72] Dahmen, J. & Cook, D. Synsys: A synthetic data generation system for healthcare applications. *Sensors* **19**, 1181 (2019).

[73] Choi, E. *et al.* Generating multi-label discrete patient records using generative adversarial networks. In *Machine Learning for Healthcare Conference*, 286–305 (PMLR, 2017).

[74] Hauke, J. & Kossowski, T. Comparison of values of pearson's and spearman's correlation coefficients on the same sets of data. *Quaestiones Geographicae* **30**, 87–93 (2011).

[75] Dash, S., Yale, A., Guyon, I. & Bennett, K. P. Medical time-series data generation using generative adversarial networks. In *18th International Conference on Artificial Intelligence in Medicine*, 382–391 (Springer, 2020).

[76] Shim, H., Lowet, D., Luca, S. & Vanrumste, B. Synthetic data generation and multi-task learning for extracting temporal information from health-related narrative text. In *Proceedings of the Seventh Workshop on Noisy User-generated Text*, 260–273 (2021).

[77] Zhang, Z., Yan, C., Lasko, T. A., Sun, J. & Malin, B. A. Synteg: a framework for temporal structured electronic health data simulation. *Journal of the American Medical Informatics Association* **28**, 596–604 (2021).

[78] Menéndez, M., Pardo, J., Pardo, L. & Pardo, M. The jensen-shannon divergence. *Journal of the Franklin Institute* **334**, 307–318 (1997).

[79] Zhang, Z., Yan, C. & Malin, B. A. Keeping synthetic patients on track: feedback mechanisms to mitigate performance drift in longitudinal health data simulation. *Journal of the American Medical Informatics Association* **29**, 1890–1898 (2022).

[80] Lahitani, A. R., Permanasari, A. E. & Setiawan, N. A. Cosine similarity to determine similarity measure: Study case in online essay assessment. In *2016 4th International Conference on Cyber and IT Service Management*, 1–6 (IEEE, 2016).

[81] Papineni, K., Roukos, S., Ward, T. & Zhu, W.-J. Bleu: a method for automatic evaluation of machine translation. In *Proceedings of the 40th Annual Meeting of Association for Computational Linguistics*, 311–318 (2002).

[82] Guan, J., Li, R., Yu, S. & Zhang, X. A method for generating synthetic electronic medical record text. *IEEE/ACM Transactions on Computational Biology and Bioinformatics* **18**, 173–182 (2019).

[83] Lee, S. H. Natural language generation for electronic health records. *NPJ Digital Medicine* **1**, 63 (2018).

[84] Plattel, C. *Distributed and incremental clustering using shared nearest neighbours*. Master's thesis, Utrecht University (2014).

[85] Rayson, P., Berridge, D. & Francis, B. Extending the cochran rule for the comparison of word frequencies between corpora. In *7th International Conference on Statistical Analysis of Textual Data*, 926–936 (2004).

[86] Zhu, Y. *et al.* Texygen: A benchmarking platform for text generation models. In *The 41st international ACM SIGIR conference on research & development in information retrieval*, 1097–1100 (2018).

[87] Lin, C.-Y. Rouge: A package for automatic evaluation of summaries. In *Text Summarization Branches Out*, 74–81 (Association for Computational Linguistics, 2004).

[88] Libbi, C. A., Trienes, J., Trieschnigg, D. & Seifert, C. Generating synthetic training data for supervised de-identification of electronic health records. *Future Internet* **13**, 136 (2021).

[89] Bandi, A., Adapa, P. V. S. R. & Kuchi, Y. E. V. P. K. The power of generative ai: A review of requirements, models, input–output formats, evaluation metrics, and challenges. *Future Internet* **15**, 260 (2023).

[90] Abell-Hart, K., Hajagos, J., Zhu, W., Saltz, M. & Saltz, J. Generating longitudinal synthetic ehr data with recurrent autoencoders and generative adversarial networks. *Data Management, Polystores, and Analytics for Healthcare* 153 (2021).

[91] Wang, Y., Meng, X. & Liu, X. Differentially private recurrent variational autoencoder for text privacy preservation. *Mobile Networks and Applications* 1–16 (2023).

[92] Reiter, J. P., Wang, Q. & Zhang, B. Bayesian estimation of disclosure risks for multiply imputed, synthetic data. *Journal of Privacy and Confidentiality* **6** (2014).

[93] Ebrahimi, Z., Loni, M., Daneshtalab, M. & Gharehbaghi, A. A review on deep learning methods for ecg arrhythmia classification. *Expert Systems with Applications: X* **7**, 100033 (2020).

[94] Khademi, S. *et al.* Data augmentation to improve syndromic detection from emergency department notes. In *Proceedings of the Australasian Computer Science Week*, 198–205 (ACM Digital Library, 2023).

[95] Syed, M., Marshall, J., Nigam, A. & Chawla, N. V. Gender prediction through synthetic resampling of user profiles using seqgans. In *8th International Conference on Computational Data and Social Networks*, 363–370 (Springer, 2019).

[96] Yoon, J., Jarrett, D. & Van der Schaar, M. Time-series generative adversarial networks. *Advances in Neural Information Processing Systems* **32** (2019).

[97] Bankó, Z. & Abonyi, J. Correlation based dynamic time warping of multivariate time series. *Expert Systems with Applications* **39**, 12814–12823 (2012).
